# *Lablab purpureus* Protects HaCaT Cells from Oxidative Stress-Induced Cell Death through Nrf2-Mediated Heme Oxygenase-1 Expression via the Activation of p38 and ERK1/2

**DOI:** 10.3390/ijms21228583

**Published:** 2020-11-14

**Authors:** Nurud Diniyah, Md Badrul Alam, Hee-Jeong Choi, Sang-Han Lee

**Affiliations:** 1 Department of Food Science and Biotechnology, Graduate School, Kyungpook National University, Daegu 41566, Korea; nurud.ftp@unej.ac.id (N.D.); mbalam@knu.ac.kr (M.B.A.); choi930302@gmail.com (H.-J.C.); 2 Faculty of Agricultural Technology, University of Jember, Jember 68121, East Java, Indonesia; 3 Food and Bio-Industry Research Institute, Kyungpook National University, Daegu 41566, Korea

**Keywords:** antioxidant, *Lablab purpureus*, heme oxygenase 1, keratinocyte

## Abstract

Ultraviolet B (UV-B) radiation induces the extreme production of either reactive oxygen species (ROS) or inflammatory mediators. The aim of this study was to evaluate the antioxidant activities of 70% ethanolic extract of *Lablab purpureus* (LPE) and the underlying mechanisms using HaCaT cells exposed to UV-B. High-performance liquid chromatography (HPLC) confirmed the presence of gallic acid, catechin, and epicatechin in LPE. LPE was shown to have a very potent capacity to scavenge free radicals. The results showed that LPE prevented DNA damage and inhibited the generation of ROS in HaCaT cells without causing any toxicity. LPE increased the expression of endogenous antioxidant enzymes such as superoxide dismutase-1 and catalase. Furthermore, LPE treatment facilitates the nuclear translocation of nuclear factor (erythroid-derived 2)-like 2 (Nrf-2), boosting the phase II detoxifying enzyme heme oxygenase-1 (HO-1) leading to the combatting of oxidative stress. However, pretreatment of LPE also caused the phosphorylation of mitogen-activated protein kinases (MAPK kinase) (p38 kinase) and extracellular signal-regulated kinase (ERK), whereas treatment with p38 and ERK inhibitors substantially suppressed LPE-induced Nrf2 and heme oxygenase (HO)-1 expression. These findings suggest that LPE exhibits antioxidant activity via Nrf-2-mediated HO-1 signaling through the activation of p38 and ERK, indicating that LPE can potentially be used as a remedy to combat oxidative stress-induced disorder.

## 1. Introduction

Several skin diseases are developed and initiated by ultraviolet (UV) irradiation [1] through the generation of reactive oxygen species (ROS) such as hydroxyl radical, superoxide anion, hydrogen peroxide, and singlet oxygen [2]. UV-B irradiation causes many detrimental effects, including deoxyribonucleic acid (DNA) and protein damage, oxidative stress, inflammation, and carcinogenesis. These events are mainly triggered by ROS and eventually lead to the development of various skin diseases [3,4]. An effective skin photoprotection strategy involves the use of antioxidants [5,6].

In recent years, accumulating evidence suggests that UV-B also influences the skin’s antioxidants, disrupting the skin’s ability to defend itself against reactive nitrogen/oxygen species [7]. However, UV-B-induced oxidative stress is certainly complemented by a unique antioxidant system engaged by the skin [8]. Even though human skin tightly regulates its intracellular redox stability, UV-B can completely destroy the epidermal protective antioxidant system, which involves superoxide dismutase (SOD), catalase (CAT), and heme oxygenase-1 (HO-1) [9].

Detoxifying enzymes of phase I and phase II are immediately highly expressed in skin cells and can quench ROS to restore cellular redox homeostasis [10]. For the upregulation of HO-1, the activation of Nrf2 is essential. Under resting conditions, Nrf2 is maintained in an inactive state in the cytoplasm due to Kelch-like ECH-associated protein 1 (Keap1). However, conformational changes in Keap1 or reactions to oxidative stress by inducers promotes the nuclear translocation of Nrf2, allowing it to bind to antioxidant response elements (ARE) and regulate the expression of various antioxidant enzymes. In addition, the activation of signaling cascades, such as mitogen-activated protein kinase (MAPK), protein kinase C (PKC), and phosphatidylinositol 3-kinase (PI3K/Akt) are essential for the nuclear translocation of Nrf2 [11,12].

A recently developed strategy for cytoprotection following UVB-induced oxidative stress is to promote the endogenous antioxidant system of the skin using components of plant-based sources [13]. *Lablab purpureus* (LP) is an important non-oil seed legume widely consumed in Indonesia, especially East Java. These legumes play an important role in human nutrition as a valuable source of plant protein. The fresh pods and seeds are eaten boiled or used as tempeh (fermented traditional food), in soup (as a vegetable), or as protein-rich flour and can be developed to be a meat substitute. The seeds contain high levels of protein, with well-balanced amino acid composition, fiber, vitamins, minerals, phytic acid, polyphenolic compounds, and antioxidants. These plants and their 70% ethanolic seed extracts exhibit potent antioxidant activities [10]. For example, the dietary consumption of whole seeds of LP can reduce ROS, which are known to be involved in UVB-induced damage. However, the effects of LP seeds on HaCaT cells and the underlying mechanisms have not been reported. Thus, UVB-induced keratinocyte models were used to explore the protective effect of a 70% ethanol extract of LP (LPE). The aim of this study was to measure the antioxidant capacity of LPE in various in vitro antioxidant assays and determine the expression levels of antioxidant enzymes in HaCaT cells.

## 2. Results

### 2.1. HPLC Analysis of LPE

**Figure 1:**
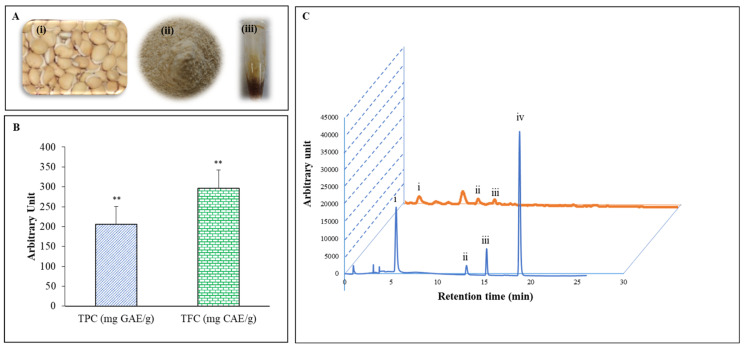
Photos of Lablab purpureus (LPE) (**A**) bean (i), powder (ii), and extract (iii). Total polyphenol content and flavonoids content (**B**) extracts of LPE (2 µL of samples). High-performance liquid chromatography (HPLC) (absorbance at 280 nm)—profile of phenolic standards and LPE (**C**). Peaks: gallic acid (i); catechin (ii); epicatechin (iii); and coumaric acid (iv). The injection volume was 20 µL, and the flow rate was maintained at 0.8 mL/min. All the tests were performed in triplicate.

Polyphenolic and flavonoids are the most important natural phenolic groups. These phenolic constituents have a wide range of chemical and biological properties, including radical scavenging activities and antioxidant properties via the quenching of singlet and triplet oxygen or decomposing peroxides [14]. Appearing of the bean, powder and extract of LPE have shown in Figure 1A. We found that polyphenols and flavonoid contents were highest in LPE with values of 205.79 ± 0.11 mg gallic acid equivalent (GAE)/g and 296.56 ± 0.01 mg catechin equivalent (CAE)/g, respectively (Figure 1B). The polyphenolic and flavonoid contents of other extracts were observed, LP distilled water >LP ethanol 100% (supplementary data Figure S1A,B). The major polyphenolics in LPE were detected using high-performance liquid chromatography (HPLC) with various standard polyphenolics. As described in Figure 1C, the chromatogram shows that LPE exhibited peaks with the same retention times as the following standard polyphenolics: gallic acid (5.536 min), catechin (13.109 min), epicatechin (15.276 min), and coumaric acid (18.822 min). The amounts of these polyphenolic compounds in LPE were analyzed by applying the peak areas of standards with known concentrations. Figure 1C shows the contents of gallic acid (2.245%), catechin (0.058%), and epicatechin (0.040%).

### 2.2. Radical Scavenging Abilities of LPE

**Figure 2:**
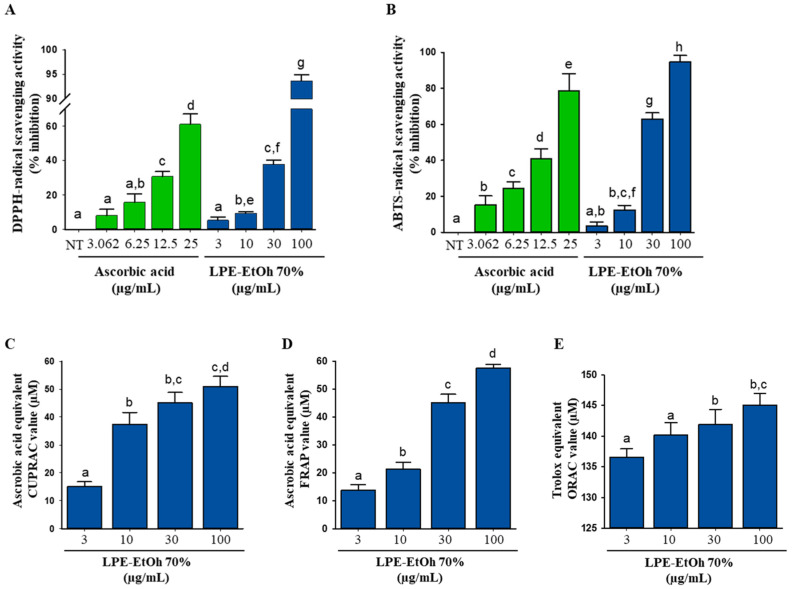
Radical scavenging effects of Lablab purpureus 70% ethanolic extract (LPE) (2 µL). The 2,2-diphenyl-1-picrylhydrazyl (DPPH)-radical scavenging assay (**A**), ABTS-radical scavenging assay (**B**), cupric reducing antioxidant capacity (CUPRAC) assay (**C**), ferric reducing antioxidant power (FRAP) assay (**D**) were performed determined concentrations of the LPE (3–100 µg/mL), and ascorbic acid were used as standard. The oxygen radical absorbance capacity (ORAC) activities of the samples were calculated by subtracting the area under the blank curve from the area under the sample curve to obtain the net area under the curve (net AUC) (**E**). Statistical values are expressed as the mean ± SD (*n* = 3). All the tests were performed in triplicate. Different letters stand for statistically significant each other (*p* < 0.05) performed by one-way ANOVA followed by Tukey’s test. Green color means ascorbic acid-treated group as a positive control and blue color means sample treated groups (LPE).

Before further research in cell lines, radical scavenging assays in cell-free systems are often performed to measure the antioxidant potential of a compound. We used 2,2’-azino-bis (3-ethylbenzothiazoline-6-sulphonic acid) (ABTS)-radical scavenging, 2,2-diphenyl-1-picrylhydrazyl (DPPH), Cupric-reducing antioxidant capacity (CUPRAC), ferric reducing antioxidant power (FRAP), and oxygen radical absorbance capacity (ORAC) assays to confirm the antioxidant ability of LPE (Figure 2A–E). These assays are widely used to evaluate the amount of an antioxidant (i.e., the antioxidant capacity for scavenging radicals) [15]. The scavenging of free radicals and ROS by donating electrons and hydrogen atoms are important mechanisms of antioxidants to directly prevent oxidative stress-induced cellular damage [16]. The DPPH scavenging activity of LPE was the highest (94.31 ± 0.11% of inhibition) at a dose of 100 μg/mL (Figure 2A). Distilled water and the 100% ethanolic extract of LP also markedly scavenged DPPH^•^ in a concentration-dependent manner (supplementary data Figure S2A). This study found that LP distilled water, 70% ethanolic extracts, and 100% ethanolic extracts were able to reduce the formation of ABTS^•+^ in the assay of electron-hydrogen atom transfer (Figure 2B and supplementary data Figure S2B). Additionally, we showed that LPE has this potential due to its electron donating ability using CUPRAC, FRAP, and ORAC assays. These results demonstrate its significant ability as a reducing power agent in a concentration-dependent manner (Figure 2C–E and supplementary data Figure S2C–E) and confirm its radical scavenging abilities.

### 2.3. Cell Viability after UV-B Exposure Following the Pretreatment of Different Concentrations of LPE

**Figure 3:**
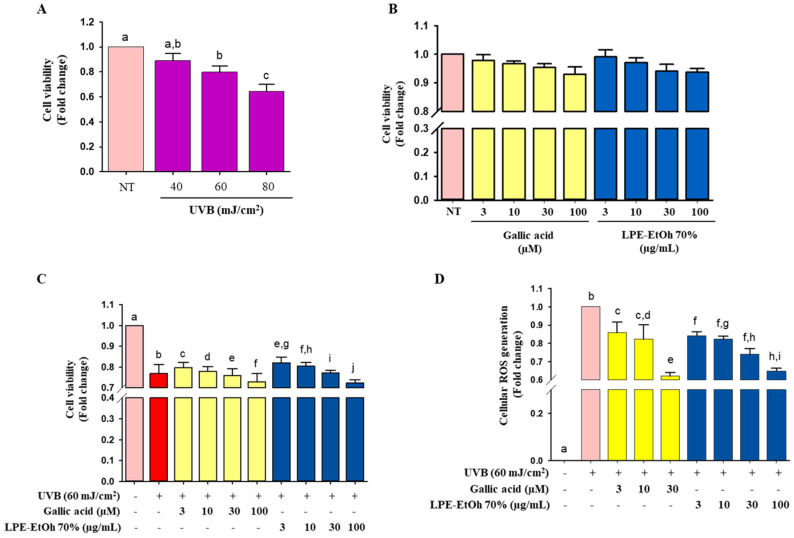
Cell viability activity of ethanolic 70% extract of Lablab purpureus L. (LPE) was evaluated by 3-(4,5-dimethylthiazol-2-yl)-2,5-diphenyltetrazolium bromide (MTT) assay. (**A**) HaCaT cells were seeded (1 × 10^5^ cells/mL) in 96-well plates for 24 h and then irradiated with UVB (40, 60, and 80 mJ/cm^2^) followed by incubation for 24 h; (**B**) HaCaT cells (1 × 10^5^ cells/mL) were treated with 2 μL of LPE ethanolic 70% extract (3–100 μg/mL) and gallic acid (3–100 μM) as a standard for 24 h. (**C**) HaCaT cells (1 × 10^5^ cells/mL) were treated with LPE (3–100 μg/mL) and GA: gallic acid (10 μM) for 24 h and then irradiated with UVB (60 mJ/cm^2^). (**D**) Pretreated HaCaT cells by LPE ethanolic 70% extract (3–100 μg/mL) and gallic acid (3–30 μM) were exposed by UVB irradiation (60 mJ/cm^2^), reactive oxygen species (ROS) levels were determined according to the Section 4. Statistical values are expressed as the mean ± SD (*n* = 3). All the tests were performed in triplicate. Different letters stand for statistically significant each other (*p* < 0.05) performed by one-way ANOVA followed by Tukey’s test. Orange color means non UV-B treated, red color means UV-B treated; yellow color means gallic acid treated; blue color meant sample (LPE) treated groups, respectively.

We next determined the effects of LPE on cell viability in HaCaT cells exposed to UV-B irradiation. Rigel et al. [16] reported that healthy highschool volunteers received the UVB irradiation daily, at 8.01 mJ/cm^2^/day. In this experiment, we used UVB radiation at 60 mJ/cm^2^, which is equivalent to approximately 7 days of sun exposure. The UV intensity was used in the analysis, the survival rate of the cells was determined by UV-B irradiation. Cells were irradiated to UV-B at various doses (0, 40, 60, and 80 mJ/cm^2^) with and without LPE and gallic acid treatment [17]. UV-B irradiation inhibited cell viability (Figure 3A). In the following experiments, the UV-B dosage was selected as 60 mJ/cm^2^ (80% of cell viability). Cell viability was measured by the 3-(4,5-dimethylthiazol-2-yl)-2,5-diphenyltetrazolium bromide (MTT) assay to evaluate the pharmaceutical effects of LPE in vitro. Gallic acid was used as a positive control for cell-based assays. LPE treatment did not show any significant cytotoxicity (Figure 3B). Thus, we fixed the concentration of LPE as 3–100 μg/mL for further cell-based experiments regarding the references [17,18]. LPE and gallic acid treatment protected the cells from the toxic effects of UV-B irradiation at concentrations of 30 μg/mL and 10 μM, respectively (Figure 3C).

### 2.4. Effect of LPE on ROS Generation

The major cause of UV-B-induced damage is an increase in ROS production, leading to oxidative stress. The spectrofluorometric analysis revealed that UV-B irradiation significantly increased intracellular ROS production in HaCaT cells (Figure 3D), but LPE treatment significantly repressed this effect in a concentration-dependent manner that was similar to gallic acid. The production of ROS in UV-B-induced cells was reduced in the presence of LPE (100 μg/mL).

### 2.5. Effect of LPE on Phase I and Phase II Antioxidant Enzyme Expression in HaCaT Cells

**Figure 4:**
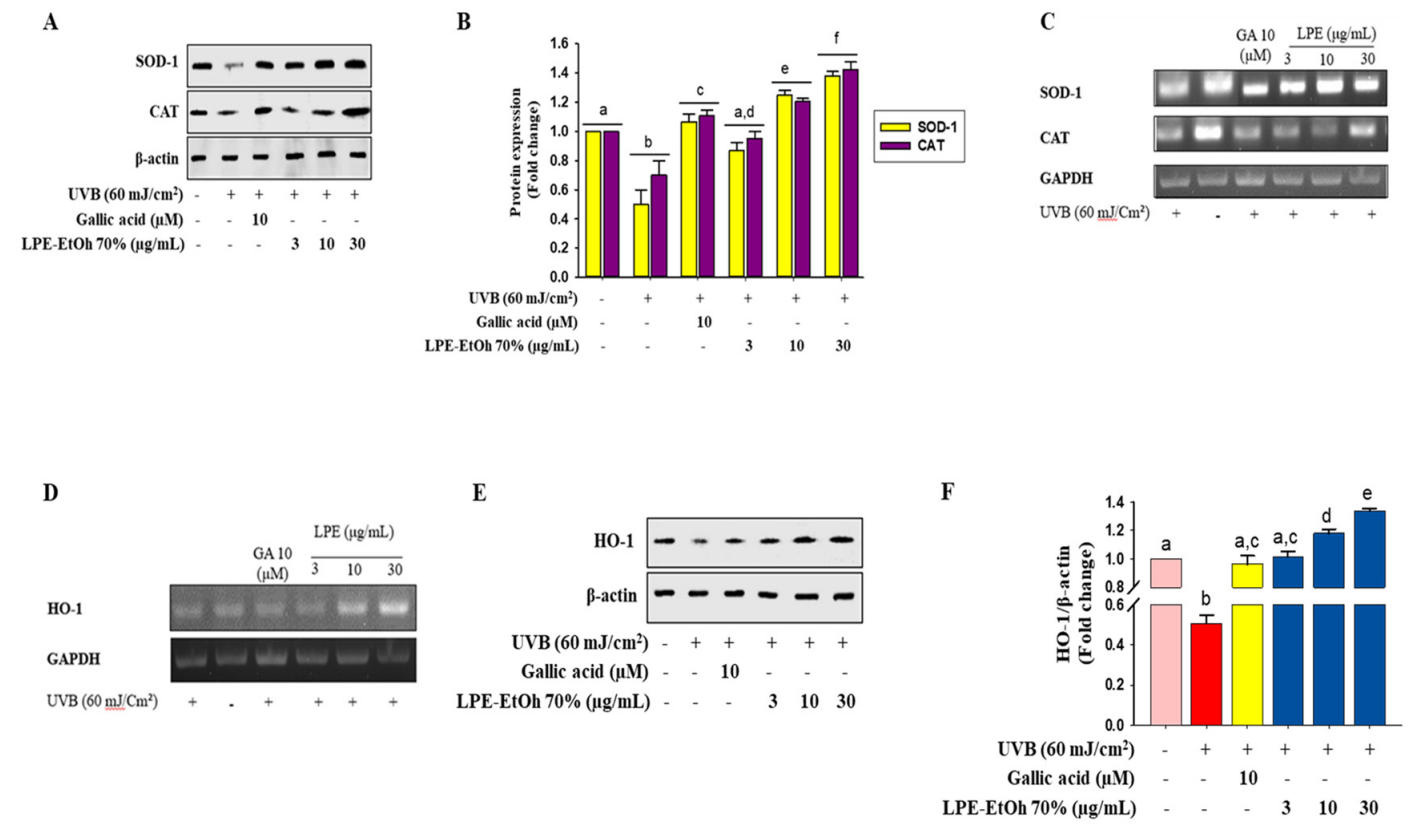
Analysis of primary and phase II antioxidant and detoxifying enzymes. HaCaT cells were pretreated with 2 µL ethanolic 70% extract of *Lablab purpureus* L. (LPE) for 24 h. Phase I antioxidant enzyme protein expression (**A**), band intensity analysis (**B**) and mRNA expression (**C**) were analyzed by Western blotting, image J software and RT-PCR, respectively. HaCaT cells were pretreated with LPE (3–30 µg/mL) for 24 h. The mRNA levels of phase II antioxidant were measured by RT-PCR (**D**). Concentration-dependent effects on HO-1 protein levels were analyzed by Western blotting (**E**) and band intensity analysis was performed by image J software (**F**). Statistical values are expressed as the mean ± SD (*n* = 3). All the tests were performed in triplicate. Different letters stand for statistically significant each other (*p* < 0.05) performed by one-way ANOVA followed by Tukey’s test. Orange color means non UVB-treatment; red color means UVB treatment, yellow color means gallic acid treatment; and blue color means sample (LPE) treatment.

This study determined the impact of LPE on the expression of primary antioxidant enzymes, such as SOD1 and catalase CAT as well as phase II detoxifying enzymes, including HO-1. HaCaT cells pretreated with various concentrations of LPE (3, 10, and 30 μg/mL). The results of immunoblotting analysis and reverse transcription-polymerase chain reaction (RT-PCR) showed that UV-B irradiation reduced the protein and mRNA expression levels of SOD and CAT. Importantly, LPE and gallic acid treatment significantly upregulated the protein levels and strongly increased their mRNA expression in a concentration-dependent manner (Figure 4A–C). Furthermore, even the mRNA expression levels of HO-1 were increased by LPE in a concentration-dependent manner, and the Western blot technique was used to confirm the enhanced protein expression of HO-1 (Figure 4C–F). Therefore, these results demonstrate an antioxidant role of LPE by modulating the expression of these enzymes.

### 2.6. Upregulation of HO-1 via Nrf2 Nuclear Translocation in HaCaT Cells

**Figure 5:**
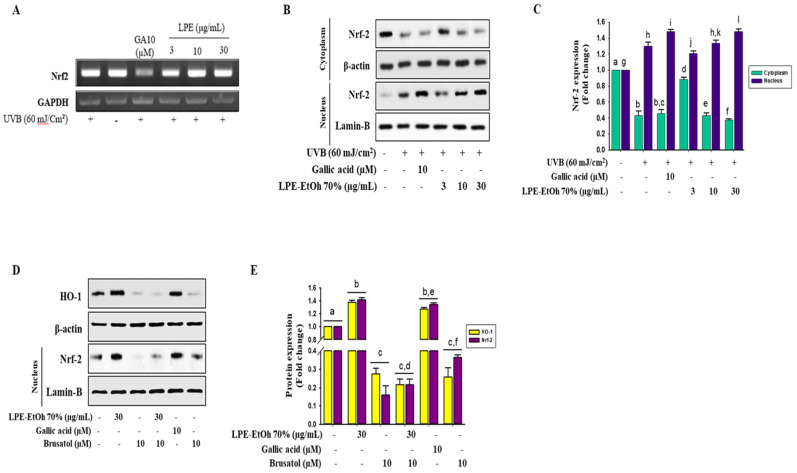
Effect of ethanol 70% extract Lablab purpureus L. (LPE) on Nrf2-mediated HO-1 expression. HaCaT cells were pretreated with 2 μL of LPE 30 μg/mL for 24 h and UVB 60 mJ/cm^2^. The mRNA expression (**A**) and protein expression of Nrf2 in cytoplasm and nucleus fractions (**B**) were analyzed by RT-PCR and Western blotting, respectively. Band intensity analysis was performed by image J software (**C**). HaCaT cells were pretreated with and without LPE 30 μg/mL, gallic acid (10 μM) and Nrf2 inhibitor (brusatol 10 μM) for 24 h and Nrf2 protein from nucleus and HO-1 protein in whole lysate were measured by Western blotting (**D**). Band intensity analysis was performed by image J software (**E**). Statistical values are expressed as the mean ± SD (*n* = 3). All the tests were performed in triplicate. Different letters stand for statistically significant each other (*p* < 0.05) performed by one-way ANOVA followed by Tukey’s test.

In the promoter regions of genes, the antioxidant response element (ARE) encodes phase II detoxifying enzymes such as HO-1 [17]. Nrf2 is released and translocate to the nucleus to upregulate the expression of phase II detoxifying enzymes in response to inducers [18]. In this study, we evaluated the mRNA expression and nuclear translocation of Nrf2 in LPE-treated HaCaT cells to elucidate the mechanism by which LPE mediates Nrf2 activation. The results showed that LPE treatment consistently enhanced the mRNA and protein level expressions of Nrf2 in a concentration-dependent manner that was comparable to gallic acid in HaCaT cells. UV-B irradiation can stimulate the cellular ROS generation in HaCat cells in our study, conformably, the UV-B induced downregulation in the protein level of HO-1 and Nrf2 (Figure 5A–C). Before LPE and gallic acid treatment, cells were treated with brusatol, a specific Nrf2 inhibitor, to determine the effectiveness of LPE in activating phase II enzymes through Nrf2-induced HO-1 expression. As predicted, brusatol inhibited Nrf2 expression, and the effects of LPE treatment were similar to gallic acid (Figure 5D,E). Additionally, the induction of HO-1 protein by LPE and gallic acid was effectively terminated following the inhibition of Nrf2 (Figure 5D,E). These results suggest that LPE can restore the antioxidant defense system via the upregulation of Nrf2-mediated HO-1 expression.

### 2.7. Effects of LPE on the MAPK Signaling Pathway

**Figure 6:**
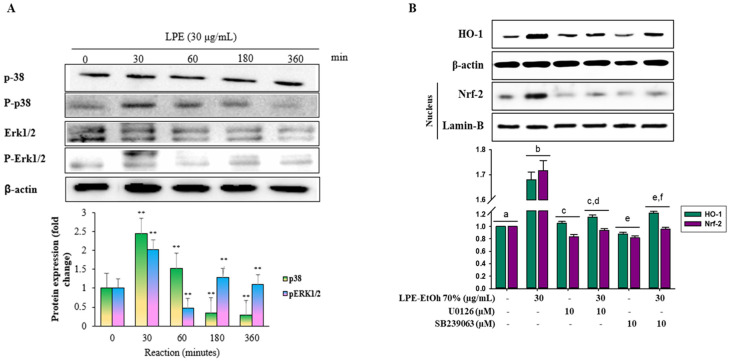
Effect of ethanol 70% extract Lablab purpureus L. (LPE) activates the translocation of Nrf2 by activating ERK1/2 and p38 pathways. HaCaT cells were pretreated with 2 μL of LPE 30 μg/mL for the indicated time and kinase activation was analyzed by Western blotting and band analysis was represented in adjacent figure (**A**). HaCaT cells were pretreated with LPE 30 μg/mL in the presence and absence of specific p38 and ERK inhibitors SB239063 (10 μM) and U0126 (10 μM), respectively and the protein levels of Nrf2 in nucleus fraction and HO-1 from whole-cell isolates were measured by Western blotting and band analysis was presented in adjacent figure (**B**). Statistical values are expressed as the mean ± SD (*n* = 3). All the tests were performed in triplicate. Different letters stand for statistically significant each other (*p* < 0.05) performed by one-way ANOVA followed by Tukey’s test.

The activation of MAPKs serves as a pivotal upstream signaling mechanism in the modulation of Nrf2 activation [19]. In this study, cells were treated with LPE (30 μg/mL) to determine the mechanisms underlying the activation of Nrf2 for an indicated time interval (30, 60, 180, and 360 min), and the phosphorylation of MAPKs ERK1/2 and p38 was assessed using Western blot analysis. Figure 6A shows that LPE treatment increased ERK1/2 and p38 phosphorylation after 30 min. However, there was no detectable c-Jun N-terminal kinase (JNK) phosphorylation in LPE-treated HaCaT cells (Figure S3). Moreover, to confirm if this upstream signaling cascade was involved in the induction of Nrf2 activity and HO-1 expression, the specific inhibitors (SB239063 for p38 and U0126 for ERK) were added to the cells treated with LPE. As predicted, inhibition of p38 and ERK1/2 pathways strongly decreased the capacity of LPE to increase the accumulation of nuclear Nrf2 and protein expression of HO-1 (Figure 6B). Based on these studies, LPE treatment enhanced the Nrf2-mediated expression of HO-1 via the activation of ERK and p38 signaling in HaCaT cells.

## 3. Discussion

During cellular metabolism, oxidative stress is induced by the production of various ROS, including superoxide (O_2_^•−^), hydroxyl (HO^•^), and hydrogen peroxide (H_2_O_2_) radicals, as toxic intracellular species. ROS-induced oxidative stress generates an imbalance between pro-oxidants and antioxidants in the production and transfer of free radicals via several cellular defense mechanisms [20,21]. ROS are chemically more reactive than O_2_^•^ and are thus produced exclusively as agents of cellular damage that can react with lipids, protein, and DNA. In this study, the antioxidant effects of LPE were evaluated using several in vitro chemical assays. It was shown that the pivotal mechanism by which LPE alleviates oxidative stress is through the transcriptional and translational regulation of a phase I oxidoreductase and phase II detoxifying enzymes via the Nrf2 pathway in HaCaT cells by activating the MAPK (p38 and ERK1/2) signaling cascade.

Flavonoids are a major group of natural compounds. They are very important natural phenolics that exhibit a wide spectrum of biological activities, including radical scavenging properties [22]. It was necessary to determine the total phenolic compounds (205.79 ± 0.11 mg gallic acid equivalent per gram of dry weight) and flavonoids (296.56 ± 0.01 mg catechin equivalent per gram of dry weight) in LPE (Figure 1B). Moreover, we compared the total phenol and flavonoid content with water and a 100% ethanolic extract (supplementary data Figure S1A,B). The 70% ethanolic extract had strong scavenging potential compared with water and the 100% extract. Polyphenolic compounds or foods high in polyphenolic content enhance the activity of SOD-1 and CAT in vitro and in vivo, resulting in a decrease in antioxidative stress [15]. Even flavonoids display antioxidant activity by inhibiting ROS/RNS producing enzymes and immediately scavenging ROS/RNS via the upregulation of antioxidant enzymes [23]. Several studies suggest that edible legumes are high in phenolic and flavonoid compounds such as gallic acid, chlorogenic acid, *p*-coumaric acid, myricetin, vanillic acid, quercetin glycosides, genistein, kaempferol, luteolin, daidzein, and tannin compounds (e.g., epicatechin, catechin, and epigallocatechin) [24,25]. Based on these previous studies, we hypothesized that polyphenolic compounds and flavonoids might be primary components of LPE. HPLC analysis was used with standard phenolic and flavonoid compounds to identify the phytochemicals in LPE. The results showed that gallic acid, catechin and epicatechin are present in LPE (Figure 1C). Previous studies have shown that polyphenolic and flavonoid compounds have anti-inflammatory, anticarcinogenic, antimicrobial, anticancer, and gastroprotective effects and can also inhibit collagenase activity [26,27,28]. A combination experiment is needed because whether a single compound or combined compounds exert these activities in our body remains unknown.

The antioxidant activity of biologically favorable compounds cannot be determined by a single method. Consequently, to investigate and understand these possible mechanisms, several antioxidant analyses, including DPPH and ABTS evaluations, as well as FRAP, CUPRAC, and ORAC assays were performed to determine the antioxidant activities of LPE. The results showed that LPE has a large range of antioxidant properties. The radical scavenging activities of LPE are shown in Figure 2. The most widely used spectrophotometric methods to confirm the antioxidant capacity of extracts or compounds are DPPH^•^ and ABTS^+•^. The free radical quenching of both DPPH and ABTS was dose-dependent and continually improved with increasing sample concentration (Figure 2A,B). These studies demonstrate that LPE has the capacity to scavenge free radicals by two different mechanisms, including a single electron transfer reaction (ABTS assay) and hydrogen transfer reaction (DPPH assay) [29].

As the antioxidant capacity is strongly associated with their reducing capacity, both FRAP and CUPRAC assays serve as a method for the measurement of antioxidant activities of various plant extracts and compounds [30], and these results are in agreement with our findings. The results provide evidence of the antioxidant potential (regarding the FRAP and CUPRAC, determined as ascorbic acid equivalent) of LPE, which was slowly enhanced with increasing concentrations of the samples (Figure 2C,D). Net AUC values of Trolox and LPE were increased in a dose-dependent manner (data not shown), indicating that LPE exhibits antioxidant activity. Furthermore, we also analyzed water and a 100% extract using DPPH, ABTS, CUPRAC, FRAP and ORAC assays (supplementary data Figure S2A−E), which indicated smaller effects than the 70% ethanolic extract. The correlation between the content of polyphenols and flavonoids and the antioxidant activity was calculated using Pearson’s coefficient r value (supplementary data Table S1). The results showed strong correlations between polyphenol and DPPH, ABTS, and FRAP assays (0.96, 0.84, and 0.86, respectively) and a moderate correlation with the CUPRAC assay (0.69). Similarly, strong correlations between flavonoids and DPPH, ABTS, and FRAP assays (0.97, 0.84, and 0.88, respectively) and a moderate correlation with the CUPRAC assay were observed (0.69). These results are similar to other studies showing correlations between polyphenol and flavonoid content with antioxidant activity [31,32,33].

The capacity to quench free radicals was defined as the direct cellular antioxidant capacity of antioxidants. ROS and RNS were quenched either by donating hydrogen or electrons, allowing protection toward oxidative stress by indirectly inducing the expression of phase II detoxifying and antioxidant enzymes [34]. ROS scavenging activity plays an important role in maintaining cellular homeostasis during cell proliferation and survival. Some enzymes such as SOD and CAT, are associated with the transfer of intracellular free radical species. Degenerative diseases can occur if the enzymes are corrupted by several incidents of oxidative stress [35]. Cells were exposed to UV-B irradiation to assess oxidative damage. Intracellular ROS are one of the most destructive products of UV-B irradiation, that induce damage to cellular macromolecules, such as proteins, mitochondrial and nuclear DNA, and lipids [36]. Several studies have shown that the pretreatment of cells with natural phytochemicals can prevent oxidative stress-induced cell toxicity [37]. Based on these reports, we pretreated HaCaT cells with LPE, which significantly decreased cell death and intracellular ROS generation induced by UV-B irradiation (Figure 3). Pastorino et al. [38] revealed experimental dose ranging from 0.25–50 µg/mL showed the ability to stimulate skin cells in order to promote tissue regeneration, prevent skin aging, and reduce fat deposition. Furthermore, cumulative studies, revealed that consumption of legumes as 20 g/day is beneficial for controlling diabetes, management of body weight, and prevention of cardiovascular diseases [39,40]. Thus, based on those previous studies, we finalized our experimental dose as 3–30 µg/mL which also had no cytotoxicity on HaCaT cells and in accordance with the previous data this dosage regimen is considered as pharmacologically active. Furthermore, LPE treatment notably enhanced both the mRNA and protein levels of antioxidant enzymes such as SOD1 and CAT in HaCaT cells (Figure 4A,B), indicating that LPE has the ability to restore cellular homeostasis and protect the cell from oxidative stress. It has been reported that SOD-1 quickly transforms O_2_^-^ into H_2_O_2_, which is eventually detoxified to H_2_O by CAT and GPX through a one-election reduction pathway, in which cytosolic superoxide radical (O_2_^−^) from O_2_ can be generated by mitochondrial electron transport chain reaction [21,35]. Previous studies supported these results and showed that some antioxidant extract treatments downregulated heat shock protein 27 (HSP27) in HepG2 and BV2 cells, thereby decreasing oxidative stress [19,26].

HO-1 produces its antioxidant effect by transforming heme into the strong pro-antioxidant biliverdin and antioxidant bilirubin that decrease with increased oxidative stress [41]. The treatment of HaCaT cells with various concentrations of LPE enhanced both the mRNA and protein expression levels of phase II detoxifying enzymes (Figure 4D–F). The antioxidant roles of several polyphenols, such as epicatechin, catechin, epigallocatechin gallate, quercetin, and caffeic acid, have been shown to involve the regulation of HO-1 expression levels to decrease cell death associated with oxidative stress [42]. Consequently, it can be assumed that the induction of phase II enzymes might contribute to the antioxidant potential of LPE to reduce oxidative stress. Accordingly, to explore this possibility, the mRNA and protein levels of Nrf2, the main regulator of phase II enzyme activation, were determined. The results showed that Nrf2 inhibition decreased the induction of HO-1 by various concentrations of LPE (Figure 5), confirming the previous study that phase II enzymes are controlled by Nrf2. Some polyphenols from another natural phytochemical extract have shown a significant ability to attenuate oxidative stress-induced liver injury through Nrf2-mediated HO-1 expression [42,43]. Other cases have shown that polyphenols activated the expression levels of Nrf2-mediated phase II enzymes in Hepa1 c1 c7, in BV2 and PC12 cells [42,44].

**Figure 7:**
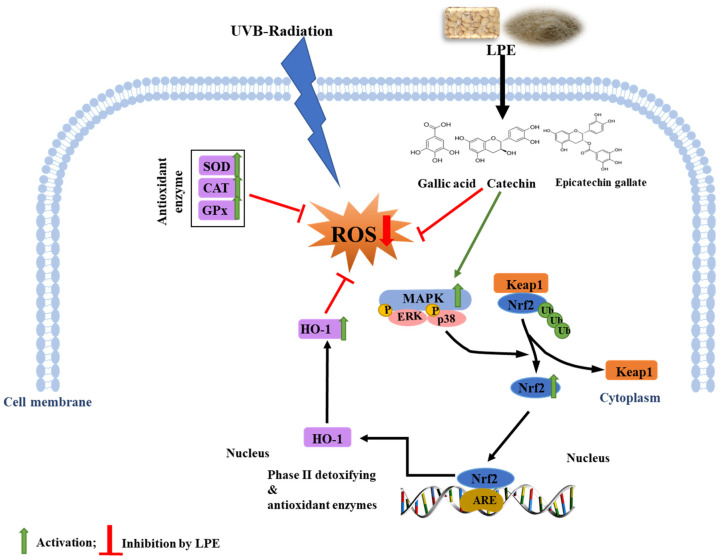
A proposed mechanism of action of 70% ethanolic extract of Lablab purpureus (LPE) against oxidative stress-induced cell death.

Diver signaling cascades, such as MAP kinase and PI3K/AKT pathways, are associated with the Nrf2/Keap1/ARE system and are known to regulate phase II gene expression [44,45]. In this study, phosphorylation of ERK1/2 and p38 by LPE was determined over time, ranging from 30 to 360 min (Figure 6A). ERK1/2 and p38 inhibitors substantially inhibited the expression of Nrf2 and HO-1 induced by LPE (Figure 6B). Therefore, the Nrf2 activation induced by LPE is dependent on the activation of ERK and p38. Currently used dietary antioxidants exhibit the ability to activate multiform cellular kinases (MAPKs and PI3K/AKT), which regulate the viability of cells in response to oxidative stress [46]. In the present study, ERK1/2 and p38 inhibitors (UO126 and SB239063, respectively) notably abolished the defensive effects of LPE on UV-B irradiation-induced cell death and ROS generation. Collectively, our results suggest that the activation of ERK1/2 and p38 pathways might be involved in the cytoprotective effects of LPE against oxidative stress (Figure 7).

## 4. Materials and Methods

### 4.1. Plant Materials and Extraction

LP (Figure 1A(i)) was obtained from Cerme village (Bondowoso district, East Java Province, Indonesia). The seeds were collected, washed, soaked in water for 1 day, peeled, cut, sun dried, dried in a hot air oven, ground to a fine powder (50-mesh), and stored at 4 °C for further studies. The powder of seeds (Figure 1A(ii) and extracts were stored in the Laboratory of Food Enzyme Biotechnology at Kyungpook National University in Daegu Korea for future reference (2019-Lpe). The powder of seeds (30 g) was mixed 10 times with 70% ethanol and placed in an ultrasonic water bath (Powersonic 420, 50/60 Hz) for 120 min at 50 °C and 40 kHz. The supernatant was collected, filtered through filter paper (No.1 Whatman Schleicher Schuell, Keene, NH, USA), and evaporated using a vacuum rotary (Eyela N-1000, Tokyo Rikakikai Co. Ltd., Tokyo, Japan). Finally, LPE was subjected to lyophilization to ensure that the extracts did not contain any trace amount of ethanol and dissolved in deionized water at a concentration of 100 mg/mL to generate a stock solution (Figure 1A(iii)). 

### 4.2. Drugs and Chemicals

DPPH, 2,2’-azino-bis (3-ethylbenzothiazoline-6-sulphonic acid), MTT, 2’,7’-dichlorofluorescin diacetate, dimethyl sulfoxide, and phosphate-buffered saline (PBS, pH 7.4) were purchased from Sigma Aldrich (St. Louis, MO, USA). Dulbecco’s modified Eagle’s medium (DMEM), fetal bovine serum (FBS), and a penicillin-streptomycin (P/S) mixture were purchased from Gibco-BRL Life Technologies (Grand Island, NY, USA). Anti-SOD1, anti-HO-1, anti-catalase, and anti-Nrf2 antibodies were purchased from Santa Cruz Biotechnology (Santa Cruz, CA, USA). Anti-phospho-p38, anti-p38, anti-ERK1/2, and anti-phospho-ERK1/2 antibodies were purchased from Cell Signaling Technology (Beverly, MA, USA).

### 4.3. HPLC Analysis

The phytochemical characterization of LPE and the standard compounds gallic acid, *p*-coumaric acid, catechin, and epicatechin were identified by HPLC-diode array detection (HPLC-DAD) with a Shimadzu Prominence autosampler (SIL-20A) HPLC system (Shimadzu, Kyoto, Japan). For reverse-phase chromatographic analysis, we used Phenomenex C18 column (4.6 × 250 mm) with 5 µm-diameter particle size. A stepwise gradient of solvent A (acetonitrile) to solvent B (1% formic acid solution) was applied by changing the ratio at every minute as follows: 10% A up to 10 min, at λ = 280 nm which was then shifted to find 30, 50, 60, 90, and 20 and 10% A in 15, 20, 25, 30, 35, and 40 min, respectively. The volume of injection was 20 µL, and the flow rate was sustained at 0.8 mL/min, as previously described [47,48]. Based on the retention time, the phenolic components were identified by comparison with the standard compounds.

### 4.4. Radical Scavenging Activity Assays

2,2’-azino-bis(3-ethylbenzothiozoline-6-sulphonic acid) (ABTS) and 2,2-diphenyl-1-picrylhydrazyl (DPPH) radical scavenging assays were used with ascorbic acid as a positive control [15]. CUPRAC and FRAP assays were used to evaluate the reducing power of LPE and the results were expressed as the ascorbic acid equivalent antioxidant value (µM) [46]. The ORAC assay was performed using Trolox as a positive control, and the potential of antioxidants was calculated as a Trolox-equivalent antioxidant value (µM) [49].

### 4.5. Cell Culture, UVB Irradiation, and Cell Viability Assay

HaCaT cells (1 × 10^5^ cells/mL) were cultured in DMEM high glucose supplemented with 10% FBS and 1% P/S at 37 °C in a 5% CO_2_ incubator. The cells in the logarithmic growth stage were treated with final concentrations of 3, 10, 30, or 100 μg/mL of LPE for 24 h, whereas control cells did not receive any vehicle or sample. The cells were exposed to 60 mJ/cm^2^ UV-B (Bio-Link Crosslinker, Vilber Lourmat, Cedex, France) set at a spectral peak of 312-nm for 20 s. Then, the cells were cultured in DMEM medium for 24 h. Cell viability was determined using the MTT colorimetric assay, as previously reported [11,48]. 

### 4.6. Measurement of Cellular ROS Generation

HaCaT cells (1 × 10^5^ cells/mL) were incubated in a 96-well plate with/without (treated as control cells) different concentrations of LPE (3, 10, 30, or 100 μg/mL) for 24 h, treated with UV-B irradiation (60 mJ/cm^2^), and then washed twice with PBS. The cells were treated with 2’,7’-dichlorofluorescein diacetate (DCF-DA) at 37 °C for 1 h in a CO_2_ incubator. The fluorescent images were obtained using a fluorescence microscope (485 nm excitation and 535 nm emission) and a fluorescence microplate reader (Victor3, PerkinElmer, Waltham, MA, USA). Intracellular ROS generation was evaluated as previously described [15], with a modification.

### 4.7. Reverse Transcription-Polymerase Chain Reaction (RT-PCR)

HaCaT cells (1 × 10^5^ cells/mL) were pretreated with different concentrations of LPE (3, 10, 30, or 100 μg/mL) for 24 h in 6-well plates. The extraction of total RNA was performed using TRIzol (Life Technologies, Gaithersburg, MD, USA) and an RT & Go Mastermix (MP Biomedicals, Seoul, Republic of Korea). Prepared complementary DNA (cDNA) served as the PCR template by using total RNA (2 μg) along with reverse transcriptase (MP Biomedicals, Santa Ana, CA, USA) and oligo (dT) primers. Various primer sequences were used to perform RT-PCR with a PCR Thermal Cycler Dice TP600 (Takara Bio Inc., Otsu, Japan) for amplification of cDNA (1 µg) (Table S2). The PCR products were subjected to 1% agarose gel electrophoresis at 100 V for 25 min, and ethidium bromide staining (Bio-Rad Laboratories, Hercules, CA, USA) was used [50]. The bands were measured by Image Lab^TM^ Software, version 5.2.1 (Bio-Rad Laboratories, CA, USA).

### 4.8. Preparation of Cytosolic and Nuclear Protein Fractionation

Cell (5 × 10^5^ cells/mL) were cultured and harvested after pretreatment with LPE at the indicated times and concentrations and pelleted by centrifugation at 280× *g* for 10 min followed by washing with 1× PBS twice. A commercially available CelLyticTM NuCLERTM extraction kit Sigma Aldrich (St. Louis, MO, USA) was used to extract the cytosolic and nuclear proteins fraction. Briefly, an ice-cold hypotonic lysis buffer 500 µL (10 mM HEPES (pH 7.9), 10 mM KCl, 1.5 mM MgCl_2_, 1 mM DTT, and 1× protease inhibitor cocktail) was used to resuspend the cell pellets. It was then incubated on ice for 15 min to allow cells to swell. After addition of 0.25% of NP-40 detergent 25 µL, the sample was vigorously vortexed for 10 s to disrupt cell membranes followed by centrifugation at 10,000× *g* for 30 s. The cytosolic fraction (supernatant) was separated from the nuclei-enriched fraction (pellet) and was stored at −80 °C. To avoid any cytosolic contamination, the nuclear fraction was washed twice with the hypotonic lysis buffer. Then a hypertonic buffer solution 25 µL (20 mM HEPES pH 7.9, 1.5 mM MgCl_2_, 0.4 mM NaCl, 25% [*v*/*v*] glycerol, 1 mM DTT, and 1× protease inhibitor cocktail) was used to extract the nuclear protein from the nuclei using with vigorous agitation for 20 min at room temperature and centrifuged at 16,000× *g* for 10 min. The final supernatant (nuclear extract) was collected and stored at −80 °C [18,50].

### 4.9. Cell Lysates and Western Blotting

Radioimmunoprecipitation assay (RIPA) buffer containing a phosphatase and protease inhibitor cocktail (10:1:1) (Sigma-Aldrich, ST. Louis, MO, USA) was used to obtain HaCaT cells (1 × 10^5^ cells/mL) lysates. The protein content in each lysate was determined using bovine serum albumin as a standard. For nuclear protein extraction, a nuclear/cytosolic fractionation kit (Sigma Aldrich, St. Louis, MO, USA) was used. The sample proteins (20 μg) were separated by 10% sodium dodecyl sulfate-polyacrylamide (SDS-PAGE) gel electrophoresis and then transferred to nitrocellulose membranes (Whatman, Dassel, Germany). The membranes were blocked in 5% skim milk for 1 h, washed with TBTS, and incubated with the indicated primary antibody overnight at 4 °C. The next day, the membranes were washed several times with TBST and then incubated with secondary antibodies (supplementary data Table S3) [51]. The bands were analyzed using Image Lab^TM^ Software, version 5.2.1 (Bio-Rad Laboratories, CA, USA).

### 4.10. Statistical Analysis

All data were analyzed by one-way analysis of variance (ANOVA) followed by Tukey’s test and presented as the mean ± standard deviation (SD; *n* = 3). Different letters of each figure stand for statistical significance (<0.05). GraphPad Prism Software (GraphPad Software, Inc., San Diego, CA, USA) was used for all statistical analyses and generating the figure. 

## 5. Conclusions

This study showed that LPE contains several polyphenolic compounds and that it exhibits strong antioxidant activity. In addition, pretreatment of HaCaT cells with LPE significantly protects the cells against UV-B induced oxidative stress by reducing cell death and decreasing ROS generation via increasing the expression of primary antioxidant enzymes and Nrf2-mediated HO-1 expression. Indeed, p38 and ERK1/2 signaling pathways were activated by LPE pretreatment. Therefore, these survival signaling cascades might be involved in the cytoprotective effect of LPE. Our research provides novel insight into the protective effects and mechanisms of *Lablab purpureus* seeds against oxidative stress.

## Supplementary Materials

The following are available online at https://www.mdpi.com/1422-0067/21/22/8583/s1.
