# Supplementary material for: Lablab purpureus Protects HaCaT Cells from Oxidative Stress-Induced Cell Death through Nrf2-Mediated Heme Oxygenase-1 Expression via the Activation of p38 and ERK1/2
Source: Int J Mol Sci. 2020 Nov 14;21(22):8583. doi: 10.3390/ijms21228583 (PMC7697790; doi:10.3390/ijms21228583)
Supplement: Supplementary file 1 [file ijms-21-08583-s001.pdf]

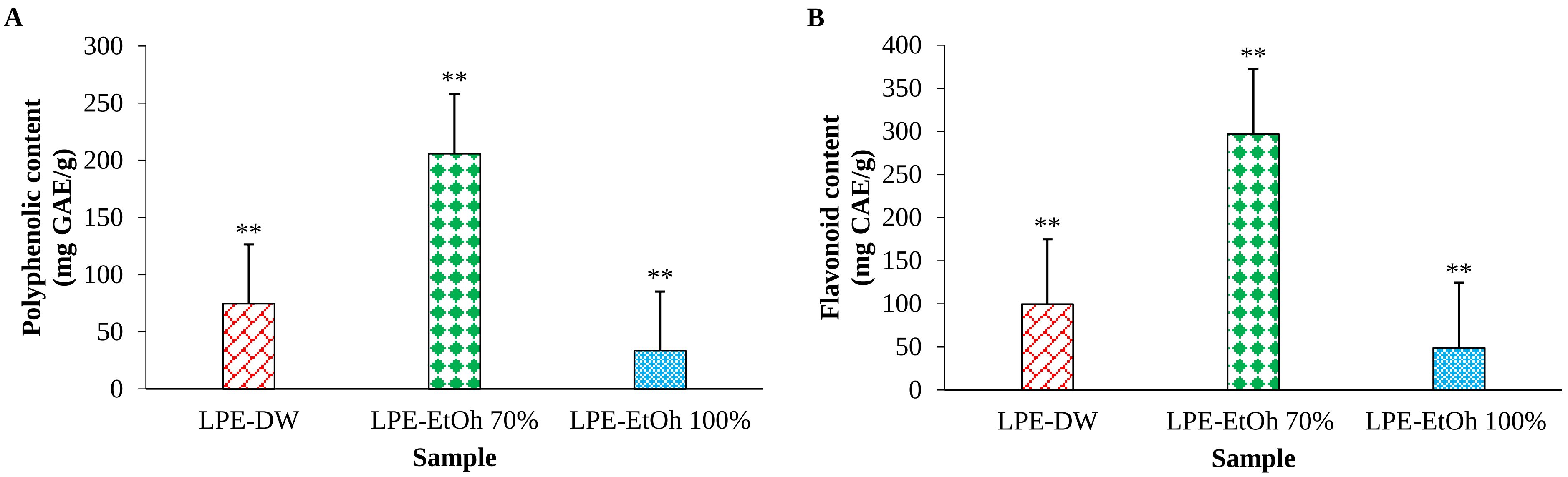

Figure S1. Total polyphenol content (**A**) and flavonoids content (**B**) 2 μL extracts of LPE in different solvent (distilled water/D.W, ethanol 70%, EtOH-70%, and ethanol 100%, EtOH-100%). Statistical values are expressed as the mean ± SD (n = 3). **p < 0.01 indicate the difference between solvent is significant. All the tests were performed in triplicate.

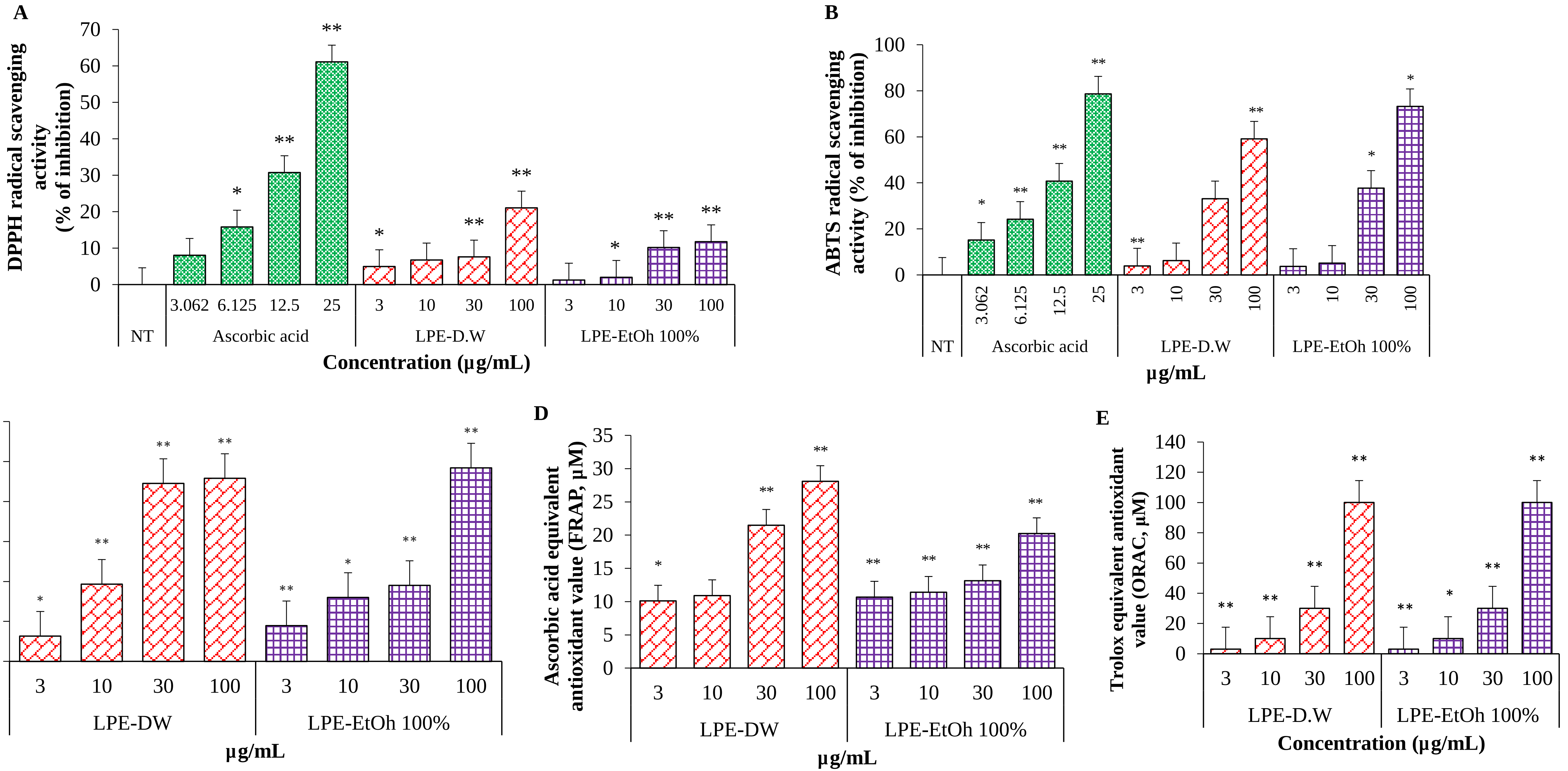

Figure S2. Radical-scavenging effects of Lablab purpureus distilled water (LPE-D.W) and 100% ethanolic extract (LPE-EtOH 100%). The 2,2-diphenyl-1-picrylhydrazyl (DPPH) radical scavenging assay (**A**), ABTS•* radical scavenging assay (**B**), cupric reducing antioxidant capacity (CUPRAC) assay (**C**), ferric reducing antioxidant power (FRAP) assay (**D**) were performed determined concentrations of the LPE, and ascorbic acid were used as standard. The ORAC activities of the samples were calculated by subtracting the area under the blank curve from the area under the sample curve to obtain the net area under the curve (Net AUC) (**E**). Statistical values are expressed as the mean ± SD (n = 3). ** $p < 0.01$ versus control using the Student's t-test. All the tests were performed in triplicate

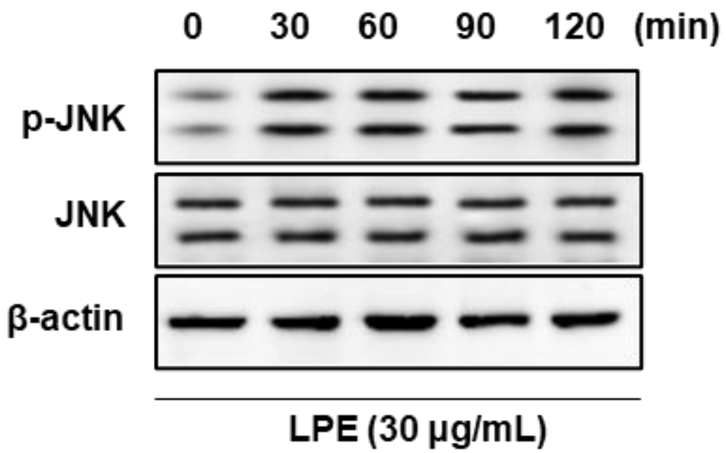

Figure S3. Effects of LPE on the activation of JNK. HaCaT cells were treated with LPE (30 µg/mL) for indicated time points and the activated and non activated forms of JNK were identified by immunoblotting assay.

Table S1. Pearson's correlation coefficient between the variables

In vitro assay	Flavonoid (R value)			Polyphenol (R value)		
	D.W	EtOh-70%	EtOh-100%	D.W	EtOh-70%	EtOh-100%
**DPPH**	0.7254	0.9675	0.6522	0.8158	0.9636	0.7251
**ABTS**	0.9677	0.8376	0.9622	0.9734	0.8432	0.8392
**CUPRAC**	0.8763	0.6982	0.968	0.8495	0.6987	0.8031
**FRAP**	0.9695	0.8842	0.9622	0.9364	0.8599	0.7848

Table S2. List of the primers used in this study

SOD1	*Forward*	*AAGCGGTGAACCAGTTGTGT*
	Reverse	*GCCAATGATGGAATGCTCTC*
CAT	*Forward*	*CACCCACGATATCACCAGATAC*
	Reverse	*GAAGACTCCAGAAGTCCCAGAC*
HO-1	*Forward*	*ACGCATATACCCGCTACCTG*
	Reverse	*TCCTCTGTCAGCATCACCTG*
Nrf-2	*Forward*	*ACATCCTTTGGAGGCAAGAC*
	Reverse	*TCGGGTCATTGTGAGTCAGT*
Gapdh	*Forward*	*GCCCAGATGGATATGGTGAA*
	Reverse	*ATGGGACGGTTCACATGTTC*

Table Supplementary Data, Diniyah et al 2020

Table S3. List of antibodies used in this study

Name	Catalog number	Company	Antigen	Host
Anti-SOD1	sc-101523	Santa Cruz Biotechnology, Inc	SOD1	Mouse
Anti-CAT	sc-515782	Santa Cruz Biotechnology, Inc	CAT	Mouse
Anti-HO-1	sc-136256	Santa Cruz Biotechnology, Inc	HO-1	Mouse
Anti *Nrf2*	sc-81342	Santa Cruz Biotechnology, Inc	Nrf2	Mouse
Anti-p-p38	sc-166182	Santa Cruz Biotechnology, Inc	p-p38	Mouse
Anti-p38	BS 3567	Santa Cruz Biotechnology, Inc	p38	Rabbit
Anti-p-ERK1/2	sc-7383	Santa Cruz Biotechnology, Inc	p-ERK1/2	Mouse
Anti-ERK1/2	BS 6472	Santa Cruz Biotechnology, Inc	ERK1/2	Rabbit
Anti-β-actin	sc-47778	Santa Cruz Biotechnology, Inc	β-actin	Mouse

Table Supplementary Data, Diniyah et al 2020
